# Evaluation of Ovarian Stimulation Initiated From the Late Follicular Phase Using Human Menopausal Gonadotropin Alone in Normal-Ovulatory Women for Treatment of Infertility: A Retrospective Cohort Study

**DOI:** 10.3389/fendo.2019.00448

**Published:** 2019-07-03

**Authors:** Xiuxian Zhu, Yonglun Fu

**Affiliations:** Department of Assisted Reproduction, Shanghai Ninth People's Hospital Affiliated to Shanghai Jiaotong University School of Medicine, Shanghai, China

**Keywords:** premature LH surge, short GnRH-a protocol, late stimulation protocol, human menopausal gonadotropin, frozen-thawed embryo transfer

## Abstract

**Objective:** To investigate the feasibility of ovarian stimulation initiated in the late follicular phase using human menopausal gonadotropin (hMG) alone in ovulatory patients undergoing *in vitro* fertilization (IVF)/intracytoplasmic sperm injection (ICSI) treatments by comparison with that of the short gonadotropin-releasing hormone agonist (GnRH-a) protocol in terms of ovarian response, embryological characteristics, and pregnancy outcomes following frozen-thawed embryo transfer (FET) cycles.

**Design:** Retrospective cohort study.

**Setting:** A university-affiliated tertiary hospital.

**Patients:** 135 infertile women undergoing their first IVF/ICSI treatment with the freeze-all strategy.

**Interventions:** In the study group, ovarian stimulation was initiated in the late follicular phase using hMG alone, with the confirmation of dominant follicular diameter ≥ 14 mm, while a short GnRH-a protocol was adopted in the control group. Oocyte maturation was induced by human chorionic gonadotropin in both groups. All good quality embryos were cryopreserved for later transfer.

**Main Outcome Measures:** The primary outcome was the incidence of premature luteinizing hormone (LH) surge. Secondary outcomes were the number of mature oocytes retrieved, good-quality embryo rate per oocyte retrieved, and clinical pregnancy rate following FET cycles.

**Results:** No premature LH surge was detected during ovarian stimulation in the study group. There was no statistically significant difference in the number of mature oocytes between the two groups (10 ± 5.6 in the study group vs. 8.51 ± 5.03 in the control group, *P* = 0.11). Good-quality embryo rate per oocyte retrieved did not differ between the two groups: 40.18% (313/779) vs. 36.67% (253/690), *P* = 0.167. Clinical pregnancy rate per transfer following FET was comparable between the two groups (61.33 vs. 52.5%, *P* = 0.267).

**Conclusions:** Our study shows that ovarian stimulation initiated in the late follicular phase using hMG alone may be a feasible alternative for normal-ovulatory women undergoing IVF/ICSI treatment with the freeze-all strategy.

## Introduction

Prevention of premature endogenous luteinizing hormone (LH) surge is of great importance during ovarian stimulation in patients with infertility undergoing *in vitro* fertilization (IVF) /intracytoplasmic sperm injection (ICSI) treatments (1). For the past several decades, when fresh embryo transfers was a routine practice in the process of IVF/ICSI, gonadotropin-releasing hormone agonist (GnRH-a), and GnRH antagonist (GnRH-ant) were the commonly-used modulators to prevent premature LH surge during ovarian stimulation (1). Nowadays, with the development of vitrificated cryopreservation techniques, frozen-embryo transfer (FET) has been widely adopted in many countries (1, 2). Alternative protocols for ovarian stimulation can therefore be considered without the constraints associated with the potential harmful effects of the hormonal environment on endometrial receptivity in combination with a freeze-all strategy (2).

As reported in our previous study (3), some patients in our tertiary-care center in China was present in different phase of the menstrual cycle when they reached hospital, owing to far distance, traffic inconvenience, insufficiency sick leave, or other personal issues. Those patients express a strong desire to commence infertility therapy as soon as possible, consequently, ovarian stimulation initiated independent of menstrual cycle is desirable. The recent observation of ovarian follicular waves in animal models as well as in humans (4) provides the foundation for explorations in flexible protocols for ovarian stimulation. In the published reports, random-start ovarian stimulation was mostly conducted in cancer patients who urgently need fertility preservation before receiving chemotherapy and/or radiotherapy on the gonads. GnRH-ant was routinely administrated for the prevention of premature LH surge when ovarian stimulation was started in the late follicular phase or luteal phase (5–7).

Our team of researchers firstly performed ovarian stimulation from the luteal phase in normal-ovulatory women, without the addition of exogenous GnRH-ant (8). Subsequently, we demonstrated cotreatment with progesterone (P) soft capsule (Utrogestan® Laboratories Besins International, France), a type of natural micronized P, was able to block premature LH surge when ovarian stimulation was commenced from the early follicular phase (9–11). Meanwhile, we confirmed the capability of dydrogesterone (Duphaston® Abbott Biologicals, Netherlands), a type of synthetic P with less metabolic burden and higher bioavailability than micronized progesterone, in the prevention of premature LH surge (12). These studies indicated that P could be used as an alternative to GnRH-a and GnRH-ant in the regulation of pituitary LH secretion during ovarian stimulation with the freeze-all strategy.

In view of those aforementioned findings, we tried to find a feasible alternative protocol to commence ovarian stimulation in the late follicular phase using human menopausal gonadotropin (hMG) alone, without exogenous pituitary modulators for LH suppression. Late follicular phase was defined as there were one dominant follicle and several subordinate follicles, and the rationale of our hypothesis was as follows: if the diameter of most subordinate follicles was <10 mm before the ovulation of the initial dominant follicle, there was no need to add exogenous pituitary modulators, whereas after ovulation, the usage of exogenous pituitary modulator was superfluous since endogenous P produced by the corpus luteum would be sufficient to prevent the occurrence of a secondary premature LH surge induced by the preovulatory follicles growing from the initial subordinate follicles (8). More importantly, the spontaneous LH surge of the initial dominant follicle would not exert any influence on subordinate follicles (<10 mm) because LH receptors of granulosa cells was less expressed in ovarian follicles with <10 mm diameter (13). So the critical issue for performing ovarian stimulation from late follicular phase without exogenous pituitary modulators was to assure the majority of subordinate follicles was <10 mm before the ovulation of the initial dominant follicle. If we presumed the diameter of the initial subordinate follicles was ~5 mm, it would take 5 days to grow to diameter ≥10 mm, on the basis of an estimated mean follicular growth rate of 1 mm per day in subordinate follicles (14, 15). Therefore, the diameter of the initial dominant follicle was calculated to be ≥14 mm to ensure the feasibility of our novel protocol when the estimated mean follicular growth rate was 2 mm per day in a dominant follicle (14, 15). In brief, we proposed ovarian stimulation initiated from late follicular phase with a dominant follicle diameter of ≥14 mm could be performed using hMG alone in the absence of exogenous pituitary modulators.

In the current study, we retrospectively collected data from normal-ovulatory patients who initiated ovarian stimulation in the late follicular phase using human menopausal gonadotropin (hMG) alone (namely late stimulation (LS) protocol) and compared the clinical outcomes with short GnRH-a protocol in terms of ovarian response, embryonic characteristics, and pregnancy outcomes following FET cycles, aiming to evaluate the feasibility of this novel protocol.

## Materials and Methods

### Study Setting and Ethical Approval

This study was conducted in the Department of Assisted Reproduction of the Ninth People's Hospital of Shanghai Jiao Tong University School of Medicine (Shanghai, People's Republic of China) which performs ~10,000 IVF cycles annually, with the approval of the hospital's Institutional Review Board. Informed consent relevant to infertility treatments with IVF/ICSI procedure was signed by all participating patients and their spouses.

### Study Design and Population

All patients included in our study were under 40 years of age with regular menstrual cycles over the past 6 months and with body mass index (BMI) of <28 kg/m^2^. In addition, the antral follicle count (AFC) in these women was >5 on menstrual cycle day (MC) 3, along with basal serum follicle-stimulating hormone (FSH) concentration <12 IU/L. Women with poor ovarian reserve (as indicated by basal FSH ≥12 IU/L or number of antral follicles <5 on ultrasound), polycystic ovarian syndrome, severe endometriosis (grade 3 or higher), or any contraindication to ovarian stimulation were excluded from the study.

This retrospective cohort study included women who underwent their first IVF/ICSI treatment with the “freeze-all” strategy from May 2016 to May 2018. The study group was treated using the LS protocol, while the short GnRH-a protocol, one of the routine regimens for patients with normal ovarian reserve in our clinic, was used in the control group. Pregnancy outcomes in FET cycles were followed up until November 2018. The choice for LS protocol in the study group was based on sufficient communication between patients and physician. Patients were aware that the stimulation protocol used is not a conventional ovarian stimulation approach and chose to use LS protocol owing personal issues, such as work schedule, business travel, a sick family member etc. In addition, only patients using LS protocol with a visit on MC 3 were included in our study.

### Ovarian Stimulation Protocol

**Figure 1:**
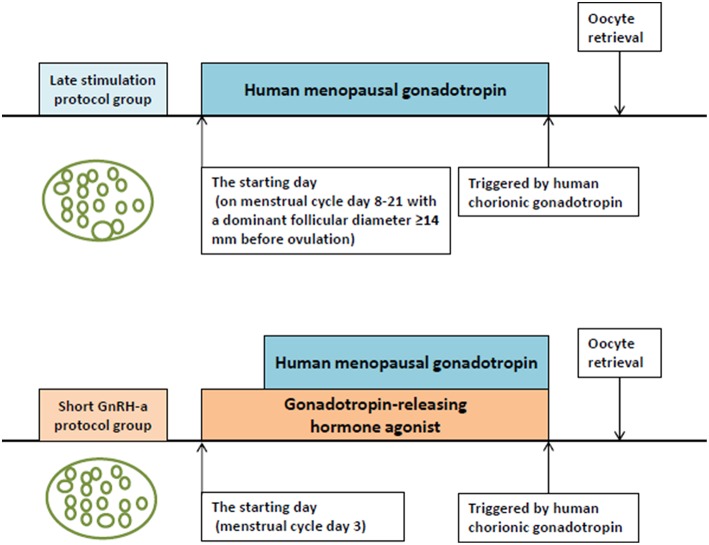
The ovarian stimulation protocols adopted in the two groups.

Figure 1 shows the ovarian stimulation protocols adopted in the two groups. The first visit of the women included in the study was on MC 3. The study group was treated using the LS protocol. The physician scheduled appointments with the patients based on results of the ultrasound examination, serum hormone levels, day of the menstrual cycle, and the estimated follicular growth rate. When the dominant follicle was measured to be larger than 14 mm by transvaginal ultrasound on MC 8–21 before ovulation, ovarian stimulation was started with the administration of hMG (Maanshan Pharmaceutical Trading Co., China) alone. The conventional short GnRH-a protocol used in the control group was as follows: 0.1 mg of GnRH-a (Decapeptyl® Ferring Pharmaceuticals, Germany) was injected daily from MC 3. On the following day, hMG administration was started using the routine method followed in our clinic for patients with normal ovarian reserve, whereby hMG was injected daily in alternating doses of 150 and 225 IU (150 IU of hMG was given on the first day of ovarian stimulation, 225 IU on the second day, again 150 IU on the third day and so on). The initiation dose of hMG was similar in both groups.

After 5–7 days, ultrasound re-examination and serum FSH, LH, E_2_, and P assays were done. Depending on the number of developing follicles as well as serum hormone concentrations, the dosage of hMG was adjusted and the next visit was scheduled. If more than three dominant follicles reached the diameter of 18 mm, 3,000 IU of hCG (Lizhu Pharmaceutical Trading Co., China) was used to induce oocyte maturation. We performed oocyte retrieval 36–38 h after hCG administration using transvaginal ultrasound-guided follicle aspiration. All follicles with diameter >10 mm were aspirated. Procedures for fertilization, embryos assessment, and vitrification were carried out as reported earlier (12).

### Endometrial Preparation and FET

Hysteroscopic screening prior to FET was recommended to detect and treat intra-uterine pathologies. If severe adhesions were diagnosed, a copper intrauterine device (IUD) (Yantai Contraceptive Instrument Co., China) was inserted for two menstrual cycles and removed by second-look hysteroscopy, which also served to ensure that intra-uterine pathologies such as recurrent adhesions were treated (16). All patients underwent endometrial preparation, the details of which have been elaborated in our earlier studies (8, 12, 16).

### Outcome Measures

The primary outcome measure was the incidence of premature LH surge. Secondary outcome measures included the number of mature oocytes, dynamic characteristics of steroid hormones, duration and dosage of hMG, number of oocytes retrieved, good-quality embryo rate per oocyte retrieved (percentage of good-quality D3 embryos and blastocysts divided by the number of retrieved oocytes) and pregnancy results following FET.

### Statistical Analysis

Data are presented as mean ± standard deviation (SD) for continuous variables and as number and percentage for categorical variables. When continuous variables were normally or near-normally distributed, Student's *t*-test was used, while Mann-Whitney *U* test was used for non-normally distributed data. The Chi square test and Fischer's exact test were adopted for categoric comparisons. Statistical significance was defined as *P* < 0.05. Data were analyzed using Statistical Package for the Social Sciences for Windows version 24.0 (SPSS, Chicago, IL, USA).

## Results

### Patient Characteristics

**Figure 2:**
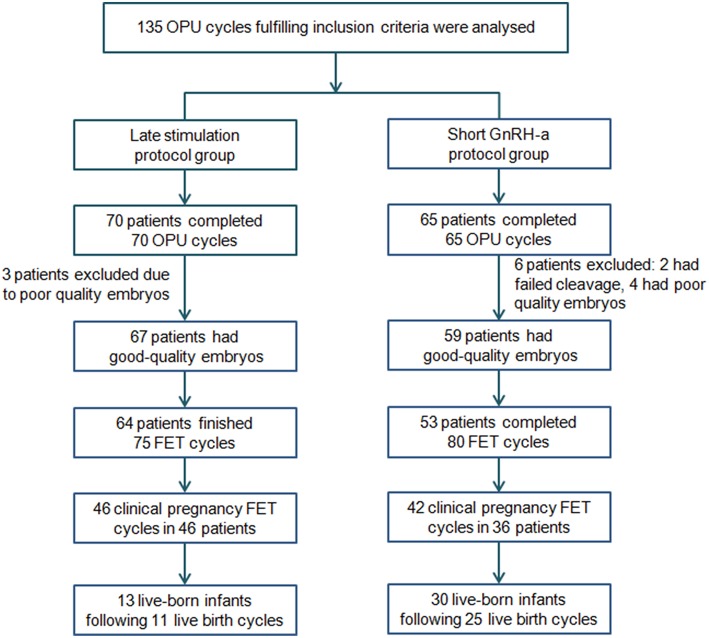
Flowchart of the study. FET, frozen-thawed embryo transfer; GnRH-a, Gonadotropin-releasing hormone agonist; OPU, oocytes pick-up.

The flowchart of the study is presented in Figure 2. A total of 135 women were eligible for analysis: 70 women in the study group and 65 in the control group. After oocyte retrieval, 3 (4.29%) patients in the study group and 6 (9.23%) in the control group did not have good-quality embryos (*P* = 0.312). In the follow-up period, 117 women completed a total of 155 FET cycles, while 9 patients with good-quality embryos have not yet finished FET. Of these, 3 belonged to the study group (one was undergoing other ovarian stimulation cycles for more embryos, and two were waiting for removal of IUD) and 6 to the control group (one got divorced, one was in another ovarian stimulation cycle, one was waiting for removal of IUD and three were due to undergo FET).

The general characteristics of patients in the study are described in Table 1. The BMI and basal P level in the control group were higher than those in the study group but these differences were not significant, with no clinical significance, while other general characteristics were comparable.

**Table 1:** General characteristics of patients undergoing *in vitro* fertilization/intracytoplasmic sperm injection treatment.

**Characteristic**	**Late stimulation protocol group (*n* = 70)**	**Short GnRH-a protocol group (*n* = 65)**	***P-*value**
Age (years), mean (SD)	31.24 (3.42)	31.71 (4.59)	0.253
Body mass index (kg/m^2^), mean (SD)	20.70 (2.26)	21.50 (2.05)	0.013
Duration of infertility (years), mean (SD)	2.84 (1.28)	2.83 (1.61)	0.690
Type of infertility, *n* (%)			0.429
Primary	34 (48.57)	36 (55.39)	
Secondary	36 (51.43)	29 (44.61)	
Antral follicle count, mean (SD)	12.89 (6.01)	12.14 (5.40)	0.535
Indication, *n*			0.082
Tubal factor	44	28	
Male factor	6	6	
Unknown factor	3	2	
Combined factors	17	29	
Basal follicle-stimulating hormone (IU/L), mean (SD)	5.56 (1.38)	5.85 (1.46)	0.211
Basal luteinizing hormone (IU/L), mean (SD)	3.16 (1.21)	3.22 (1.43)	0.894
Basal estradiol (pg/mL), mean (SD)	37.62 (16.81)	34.54 (13.85)	0.335
Basal progesterone (ng/mL), mean (SD)	0.32 (0.13)	0.26 (0.14)	0.040

*GnRH-a, Gonadotropin-releasing hormone agonist*.

### Dynamic Hormone Levels During Ovarian Stimulation

**Figure 3:**
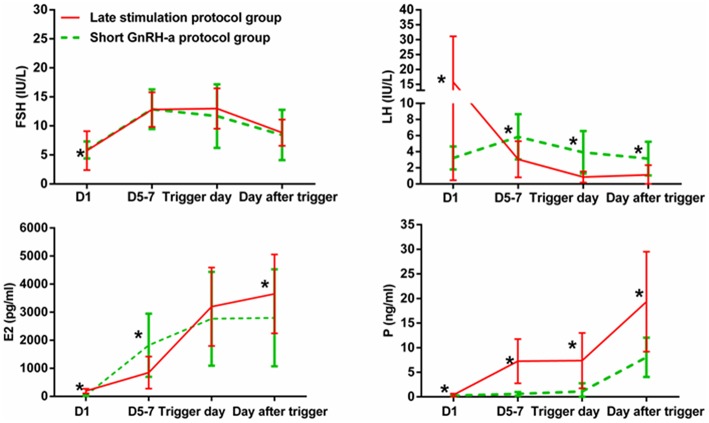
Serum hormone profiles during ovarian stimulation in the two groups. The green lines represent the control group (short GnRH-a protocol group), and the red lines represent the study group (late stimulation protocol group). The day of starting ovarian stimulation is denoted as day 1 (D1). FSH, follicle-stimulating hormone; LH, luteinizing hormone; E_2_, estradiol; P, progesterone. ^*^time point at which *P* < 0.05.

Figure 3 shows serum hormone concentrations of FSH, LH, E_2_, and P in the two groups. The starting day was denoted as day 1 (D1). None of the patients in the study group had secondary premature LH surges excluding the spontaneous ovulating LH surge of the leading follicle. In the study group, average E_2_ on D5-7 was lower than that in the control group and later increased above levels in the control group on the day after the trigger (*P* < 0.05). Serum P in the study group was significantly higher than in the control group at every determination point (*P* < 0.05).

Ovulation was not observed in four patients of the study group without increase in serum P (0.5–1 ng/mL). The number of good-quality embryos was 1, 3, 4, and 5, respectively, in these four patients. The patient with one good-quality embryo underwent a repeated cycle for oocyte retrieval, one patient had term twin delivery, one was pregnant with single gestation sac, and one had early miscarriage.

### Ovarian Stimulation Characteristics and Embryological Outcomes

Table 2 shows ovarian stimulation characteristics and embryological outcomes in both groups. Total hMG dose (2118.21 ± 351.19 vs. 1652.31 ± 562.18 IU) was greater and mean hMG duration (10.93 ± 1.65 vs. 8.57 ± 2.06) was longer in the study group than in the control group (*P* < 0.001). The number of follicles with diameter >14 mm on the trigger day and the number of follicles with diameter >10 mm on oocyte retrieval day were similar between the two groups. Number of mature oocytes was 10 ± 5.6 in the study group and 8.51 ± 5.03 in the control group (*P* = 0.11). Similarly, the two groups were comparable regarding number of oocytes retrieved, fertilized oocytes, cleaved embryos, and good-quality embryos. Additionally, good-quality embryo rate per oocyte retrieved showed no significant differences between the two groups.

**Table 2:** Ovarian stimulation characteristics and embryological outcomes of ovarian stimulation in the two groups.

**Characteristic**	**Late stimulation protocol group (*n* = 70)**	**Short GnRH-a protocol group (*n* = 65)**	***P-*value**
Duration of human menopausal gonadotropin (days), mean (SD)	10.93 (1.65)	8.57 (2.06)	<0.001
Dose of human menopausal gonadotropin (IU), mean (SD)	2118.21 (351.19)	1652.31 (562.18)	<0.001
No. of follicles with diameter >14 mm on trigger day, mean (SD)	11.71 (6.19)	7.91 (3.39)	0.184
No. of follicles with diameter >10 mm on oocyte retrieval day, mean (SD)	15.34 (8.36)	14.08 (7.29)	0.410
No. of oocytes retrieved, mean (SD)	11.13 (5.93)	10.62 (6.32)	0.521
No. of mature oocytes, mean (SD)	10.00 (5.60)	8.51 (5.03)	0.110
No. of fertilized oocytes (2PN), mean (SD)	8.04 (4.42)	7.32 (4.62)	0.250
No. of cleaved embryos, mean (SD)	7.97 (4.40)	6.95 (4.55)	0.153
No. of good-quality Day 3 embryos, mean (SD)	3.61 (2.31)	2.91 (2.29)	0.065
No. of all good-quality embryos (Day 3 embryos and blastocysts), mean (SD)	4.47 (2.55)	3.89 (2.98)	0.118
Good-quality embryo rate per oocyte retrieved, % (no. of good-quality embryos/no. of oocytes retrieved)	40.18 (313/779)	36.67 (253/690)	0.167

*GnRH-a, Gonadotropin-releasing hormone agonist, 2PN, two pronuclei*.

### Pregnancy Outcomes Following Frozen-Thawed Embryo Transfer

As illustrated in Table 3, the method of endometrial preparation and number of transferred embryos were comparable between the two groups. Clinical pregnancy rate per transfer (61.33 vs. 52.5%, *P* = 0.267) and implantation rate (40.71 vs. 34.72%, *P* = 0.297) were higher in the study group than in the control group, though the differences were not significant. There were no significant differences in the rates of biochemical pregnancy, multiple pregnancy, or early miscarriage. Birth weights of singleton newborns and twin newborns were comparable between the two groups.

**Table 3:** Pregnancy and live birth outcomes of frozen-thawed embryos in the two groups.

**Outcomes**	**Late stimulation protocol group**	**Short GnRH-a protocol group**	***P-*value**
No. of patients, *n*	64	53	
No. of FET cycles, *n*	75	80	
No. of transferred embryos, mean (SD)	1.87 (0.342)	1.80 (0.403)	0.268
Endometrium preparation, *n*			0.409
Natural cycle	44	47	
Stimulated cycle	26	23	
Hormone replacement therapy	5	10	
**PREGNANCY OUTCOME OF FET**			
Biochemical pregnancy rate per transfer, % (no. of FET cycles with positive serum hCG results /no. of all the FET cycles)	64.00 (48/75)	55.00 (44/80)	0.254
Clinical pregnancy rate per transfer, % (no. of intrauterine and ectopic pregnancy cycles with or without fetal heart activity /no. of all the FET cycles)	61.33 (46/75)	52.50 (42/80)	0.267
Multiple pregnancy rate, % (no. of FET cycles with more than one gestational sac/no. of all the clinical pregnancy cycles)	26.09 (12/46)	21.43 (9/42)	0.609
Implantation rate, % (no. of gestation sac /no. of embryos transferred)	40.71 (57/140)	34.72 (50/144)	0.297
Ectopic pregnancy rate, % (no. of FET cycles with ectopic pregnancy /no. of all the clinical pregnancy cycles)	2.170 (1/46)	2.38 (1/42)	0.962
Early miscarriage rate % (no. of FET cycles with empty gestational sac or stopped fetal heart before the gestational age of 12 weeks / no. of all the clinical pregnancy cycles)	6.52 (3/46)	14.29 (6/42)	0.300
**LIVE-BIRTH OUTCOMES**			
Live birth cycle, *n*	11	25	0.699
Term delivery	9	19	
Preterm delivery	2	6	
New-born			0.869
Single birth, *n*	9	20	
Twin birth, *n*	4	10	
Single birth weight (g), mean (SD)	3532.22 (478.82)	3157.50 (669.41)	0.199
Twin birth weight (g), mean (SD)	2250.00 (227.30)	2480.00 (356.06)	0.374

*FET, frozen embryo transfer; GnRH-a, Gonadotropin-releasing hormone agonist*.

## Discussion

To the best of our knowledge, our study firstly explored the switch to endogenous progesterone in place of conventional exogenous formulations to suppress LH surge during late follicular phase-start ovarian stimulation. As revealed by dynamic serum hormone determination, no secondary premature LH surge was observed in the study group. These data provides evidence that ovarian stimulation initiated in the late follicular phase could be performed using hMG alone in the absence of exogenous pituitary modulators, without the constraints of premature LH surge. The comparable ovarian response, embryological characteristics, and pregnancy outcomes in FET cycles between the two groups implied that this novel protocol was effective in producing competent oocytes/embryos and therefore, was non-inferior to the conventional short GnRH-a protocol.

Performing ovarian stimulation from the late follicular phase is not new, which was earlier generally used in women recently diagnosed with cancer and due for gonadotoxic therapy. In a retrospective study, Cakmak et al. commenced ovarian stimulation when a dominant follicle was confirmed to have diameter >13 mm and serum P level <2 ng/ml after MC 7 (5). They recommended using GnRH-ant from the beginning of ovarian stimulation until triggering if the follicles following the lead follicle reached 12 mm before spontaneous LH surge, whereas, if the follicles were <12 mm and stayed so before spontaneous LH surge, they believed ovarian stimulation could be started without GnRH-ant. After LH surge, GnRH-ant were to be employed when the secondary follicles reached 12 mm, to prevent premature secondary LH surges (5). In another study, Qin et al. tried initiating ovarian stimulation in the late follicular phase in infertility patients receiving IVF/ICSI treatment in combination with FET (3), whereby GnRH-a and hMG were administered concomitantly. This was followed by oral medroxyprogesterone acetate and clomiphene citrate from the following day onward, when the dominant follicle diameter reached >10 mm and serum E_2_ level >75 pg/mL on MC 6–14 (3). However, it seems to be unnecessary to add exogenous progestational agents and GnRH-ant after the rupture of the initial dominant follicle in ovarian stimulation initiated in the late follicular phase because the endogenous P secretion following spontaneous ovulation is able to achieve consistent LH suppression, as reported in our earlier studies (3, 8) and previous animal model experiments (17, 18).

The concept of the LS protocol in our study differs from previously described ovarian stimulation protocols started in late follicular phase. In our preliminary assumption, the dominant follicle of patients in the study group would rupture spontaneously without induction by GnRH-a. However, spontaneous ovulation was not observed in four patients on the LS protocol, and interestingly, they showed consistent LH suppression by autologous regulation without premature luteinization and early ovulation. This phenomenon was a common case in the classical gonadotropin stimulation procedure. Probability of occurrence of premature LH surges is estimated at 20% during ovarian stimulation from the early follicular phase using hMG alone (1, 19), indicating that the addition of pituitary modulators maybe unnecessary in some patients, though underlying mechanisms and reliable indicators remain obscure. Those cases show that even if the initial dominant follicle has not ovulated when ovarian stimulation has been started for several days and the diameter of most subordinate follicles has surpassed 10 mm, it was possible to perform ovarian stimulation without the addition of exogenous pituitary modulator. That is to say, the late follicular phase with a dominant follicle diameter of ≥14 mm on MC 8–21 before spontaneous ovulation, may be a starting window in normo-ovulatory women, from which ovarian stimulation can be completed using hMG alone in the absence of exogenous pituitary modulator. Nevertheless, additional clinical studies in a large scale are still needed to assess the efficacy of this flexible regimen, and identify the optimal time point for the initiation of late follicular phase ovarian stimulation on the basis of the diameter of follicle, serum hormone levels, or other relevant indicators.

Though the application of the multi-follicular-waves theory in ovarian stimulation is not new, potential negative effects of P on follicle development and quality of oocytes/embryos are still major concerns, due to which initiation of ovarian stimulation from the late follicular phase is but an alternative approach in routine IVF procedure. Cakmak et al. revealed that oocytes retrieved from cancer patients following ovarian stimulation started in the late follicular phase and luteal phase showed early developmental competence comparable to those derived from the conventional protocol started in the early follicular phase (4). Similarly, Qin et al. observed no statistically significant differences in embryological characteristics of patients in whom ovarian stimulation was initiated in the early-late follicular or luteal phase (3). Moreover, the pregnancy and live-birth outcomes in patients who underwent ovarian stimulation in the luteal phase were similar to those of patients treated with the short GnRH-a protocol (20). In our earlier studies, we have corroborated that rates of oocyte retrieval, good-quality embryos, clinical pregnancy, and live births are comparable between patients in whom ovarian stimulation was started in the early follicular phase using exogenous P administration for LH suppression and in those patients in whom the short GnRH-a protocol was used (9, 21). These studies provide sound evidence that neither folliculogenesis nor the developmental potential of the oocyte/embryo were compromised as a result of endogenously produced P by the corpus luteum and exogenously administered P agents in the early-late follicular phase. These findings are in line with those of our current study, wherein we found no statistically significant difference in the number of mature oocytes, good-quality embryo rate per oocyte retrieved, and clinical pregnancy rate between the two groups. These data confirmed that the oocytes and embryos resulting from the LS protocol showed developmental potential similar to that of those derived from the short GnRH-a protocol.

The retrospective design and relatively small sample size are major limitations of this study. In addition, some patients did not complete FET cycles despite having good-quality embryos due to various reasons. Also, the intervals of hormonal analyses and follicle measurement were inconsistent, which may have contributed to bias. Therefore, the current study must be considered a preliminary step warranting generation of further evidence by larger prospective studies to confirm our findings and explore the efficacy, optimal initiation point, and long-term safety of this novel protocol before offering it routinely as a part of IVF/ICSI.

## Conclusion

Our study shows that ovarian stimulation initiated in the late follicular phase could be performed using hMG alone without the constraints of premature LH surge. We also demonstrated the novel protocol obtained similar ovarian, embryological, and clinical outcomes as that of the short GnRH-a protocol. These results may provide physicians with an effective alternative in the management of ovarian stimulation in combination with embryo cryopreservation for infertility treatment and fertility preservation. However, in the light of these findings of our retrospective cohort study with a small sample, we recommend this protocol to be further evaluated by larger studies with prospective design to evaluate its effectiveness in routine infertility practice.

## Data Availability

The raw data supporting the conclusions of this manuscript will be made available by the authors, without undue reservation, to any qualified researcher.

## Ethics Statement

This study was conducted in the Department of Assisted Reproduction of the Ninth People's Hospital of Shanghai Jiao Tong University School of Medicine (Shanghai, People's Republic of China) with the approval of the hospital's Institutional Review Board. Informed consent relevant to infertility treatments with IVF/ICSI procedure was signed by all participating patients and their spouses.

## Author Contributions

XZ collected the data and wrote the manuscript. YF designed the study and revised the manuscript.

### Conflict of Interest Statement

The authors declare that the research was conducted in the absence of any commercial or financial relationships that could be construed as a potential conflict of interest.
